# The Microbiota of Modified-Atmosphere-Packaged Cooked Charcuterie Products throughout Their Shelf-Life Period, as Revealed by a Complementary Combination of Culture-Dependent and Culture-Independent Analysis

**DOI:** 10.3390/microorganisms9061223

**Published:** 2021-06-04

**Authors:** Evelyne Duthoo, Geertrui Rasschaert, Frédéric Leroy, Stefan Weckx, Marc Heyndrickx, Koen De Reu

**Affiliations:** 1 Fisheries and Food (ILVO)—Technology and Food Science Unit, Flanders Research Institute for Agriculture, 9090 Melle, Belgium; evelyne.duthoo@ilvo.vlaanderen.be (E.D.); Geertrui.Rasschaert@ilvo.vlaanderen.be (G.R.); marc.heyndrickx@ilvo.vlaanderen.be (M.H.); 2 Research Group of Industrial Microbiology and Food Biotechnology (IMDO), Faculty of Sciences and Bioengineering Sciences, Vrije Universiteit Brussel, 1050 Brussels, Belgium; frederic.leroy@vub.be (F.L.); stefan.weckx@vub.be (S.W.); 3 Department of Pathology, Bacteriology and Avian Diseases, Ghent University, 9820 Merelbeke, Belgium

**Keywords:** cooked ham, cooked chicken, modified atmosphere packaging, shelf life, spoilage

## Abstract

Although refrigeration and modified-atmosphere packaging (MAP) allow for an extended shelf life of cooked charcuterie products, they are still susceptible to bacterial spoilage. To obtain better insights into factors that govern product deterioration, ample information is needed on the associated microbiota. In this study, sliced MAP cooked ham and cooked chicken samples were subjected to culture-dependent and culture-independent microbial analysis. In total, 683 bacterial isolates were obtained and identified from 60 samples collected throughout the storage period. For both charcuterie types, lactic acid bacteria (LAB) constituted the most abundant microbial group. In cooked ham, *Brochothrix thermosphacta* was highly abundant at the beginning of the shelf-life period, but was later overtaken by *Leuconostoc carnosum* and *Lactococcus piscium*. For cooked chicken products, *Latilactobacillus sakei* was most abundant throughout the entire period. Additionally, 13 cooked ham and 16 cooked chicken samples were analyzed using metabarcoding. Findings obtained with this method were generally in accordance with the results from the culture-dependent approach, yet they additionally demonstrated the presence of *Photobacterium* at the beginning of the shelf-life period in both product types. The results indicated that combining culture-dependent methods with metabarcoding can give complementary insights into the evolution of microorganisms in perishable foods.

## 1. Introduction

According to estimates by the United Nations Environment programme, approximately 931 tons of food waste were generated in 2019, which suggests that 17% of global food production may be wasted [1]. For a large part, this is due to food spoilage [2]. Meat accounts for approximately 4% of total food waste [3], with meat losses being severest at the end of the food chain in industrialized regions. Here, almost half of meat waste occurs at the consumption level [2].

Spoilage of meat is usually of microbial origin, which is also the case for the specific category of cooked meat products. The latter are rich in proteins, fats and some (added) carbohydrates which can be broken down and metabolized by specific spoilage organisms (SSOs), resulting in off-flavors, off-odors, discoloration, gas formation, changes in texture and slime-formation, making the product unsuitable for consumption [4,5,6,7]. Although cooking eliminates the majority of the SSOs, products are recontaminated during handling operations after the cooking process, for instance during slicing and/or packaging. Microbial surface contamination of the cooked meat products thus affects the potential shelf-life stability [8,9,10], creating important food preservation challenges [11].

Refrigeration and modified-atmosphere packaging (MAP) allows for an extension of the shelf-life period of cooked meat products with minimal changes in the physical and chemical properties of the product [12,13]. Growth of psychrophilic and psychrotolerant lactic acid bacteria (LAB) and *Brochothrix thermosphacta* is generally favored under anaerobic MAP conditions with N_2_ and CO_2_. Due to competitive effects, the growth of acid-sensitive Gram-negative bacteria is largely inhibited [14,15,16,17]. Though LAB are themselves responsible for sensory changes, these changes are only presented once the stationary growth rate is approached, resulting in a product that is consumable for a period of approximately three weeks [8].

The end of the shelf-life period is defined by a maximum acceptable level of either SSOs or sensory alterations in the product [6]. Therefore, it is impossible to predict the shelf life without sufficient information on the composition of the microbiota present. Merely characterizing LAB as a group is insufficient, however, as more fine-grained information on their diversity is needed; specific species and even strains have different abilities to spoil meat [6,8,9].

In the present study, the microbiota of two types of MAP sliced cooked charcuterie (i.e., cooked ham and cooked chicken), produced at different production sites but sliced in the same facility, were investigated during storage using conventional culture-dependent microbiological analysis in combination with 16S rRNA gene metabarcoding during the shelf-life period. The metabarcoding approach was included to generate a culture-independent and cost-effective approach to obtain a complementary overview of the microbial composition of the samples on different taxonomic levels, although underrepresented communities may be overlooked [18,19,20]. The study aimed to yield additional insights into the diversity and evolution of the most abundant microorganisms that are present in these products during their shelf-life period. More specifically, the purpose was to unravel the differences and similarities between the microbial consortia that are present on two different charcuterie products undergoing similar processing steps of cooking, packaging, and slicing, but derived from different meats (in casu, pork and poultry).

## 2. Materials and Methods

### 2.1. Sample Collection and Storage

**Figure 1:**
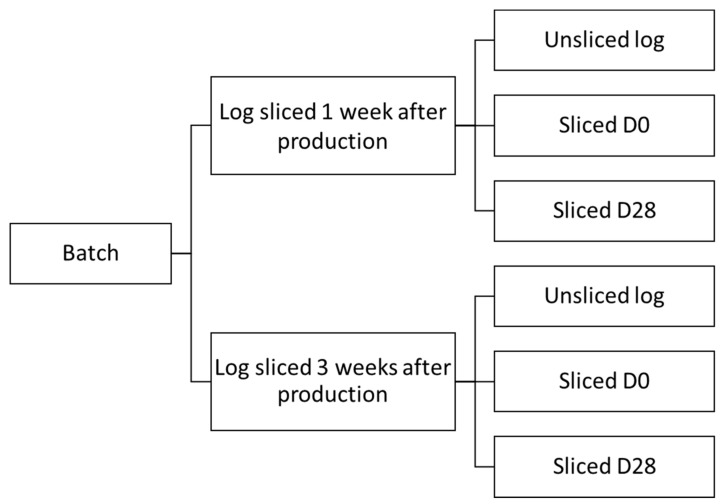
Schematic overview of sample collection procedure, resulting in six samples per batch. This scheme is applicable for each of the product types (per product type, five batches were analyzed).

Two types of food products were sampled in this study: (1) cooked ham and (2) cooked chicken products. Both products were pressed into meat logs and underwent a cooking process wherein cooked ham reached a core temperature of approximately 69 °C, whereas cooked chicken reached at least 72 °C. The samples were collected within the premises of the food business operator (FBO), at the moment of slicing and packaging. Samples were taken aseptically of the log of each product type, right before slicing occurred, as well as of the sliced products derived thereof. The logs had a shelf-life period of three weeks each, and were sliced by the FBO between one and three weeks of storage at −1.5 °C, depending on the market demand. To reflect this variability, each batch of each product type was sampled twice. This was done when the log was sliced one week after its production and when another log of the same batch was sliced two weeks later, at the end of the log’s shelf-life period. A schematic overview of the sample collection procedure is presented in Figure 1.

All sliced products derived from the various logs were packaged under MAP conditions of 70% N_2_ and 30% CO_2_. Each sliced MAP product was analyzed on the day of slicing (D0) as well as after 28 d of storage (D28), corresponding with the end of the shelf-life period of the sliced product. The 28-d storage period consisted of 19 d at 7 °C and an additional 9 d at 8 °C, per usual practice of storage studies by the FBO.

Five batches were analyzed per product type, resulting in 30 analyzed samples per product type or 60 analyzed samples in total. Samples were stored and transported under cooled conditions to the laboratory for further analysis (with a transport duration of maximum 30 min).

An additional environmental sampling was conducted eight months after the original samplings. Environment samples were taken of food contact surfaces from two slicers during production; one slicer was used to slice cooked ham and another one to slice cooked chicken. Surface sampling (approximately 625 cm^2^ each; A4 format) was performed using sponge sticks (3M™, St. Paul, MN, USA), moistened with 10 mL of maximum recovery diluent (MRD, Oxoid, Basingstoke, Hampshire, UK).

### 2.2. Microbiological Analysis

Ten grams of each food product sample were subsampled and analyzed. After preparing appropriate dilutions in MRD, the following microbiological enumerations were performed: total psychrotolerant and psychrophilic aerobic (TAC) and anaerobic microbial counts (TANC), psychrotolerant and psychrophilic lactic acid bacteria (LAB), *Enterococcus* spp., Enterobacterales, sulfite-reducing *Clostridia* spp., *Bacillus cereus*, *Brochothrix thermosphacta*, and yeast and moulds. Plate count agar (PCA; Oxoid) was used for the enumeration of TAC, applying incubation at 21 °C during 5 d. Reinforced clostridial agar (RCA; Oxoid) was incubated at 21 °C during 5 d for the enumeration of TANC. For the enumeration of presumptive LAB, de Man, Rogosa and Sharpe agar (MRS; Oxoid) and M17 agar (Oxoid) were both incubated at 21 °C during 5 d. This was done to obtain an enhanced overview of the LAB present, as MRS medium contains acetate, whereas M17 does not. Acetate inhibits growth of *Carnobacterium* spp., which is known to occur in meat products [14]. For the specific enumeration of enterococci within the LAB group, Slanetz and Bartley agar (Oxoid) were incubated at 37 °C for 48 h. The confirmation of enterococci was performed by testing for growth at 44 °C, testing for growth in 40% bile and performing a catalase test. Violet-red-bile-glucose agar (VRBG; Bio Rad, Marnes-la-Coquette, France) was incubated at 37 °C for 24 h for the enumeration of presumptive Enterobacterales. Tryptose sulphite cycloserine agar (TSC; Oxoid) was incubated at 37 °C for 24 h for the enumeration of sulfite-reducing clostridia. Mannitol egg yolk polymyxin agar (MYP; Oxoid) was incubated at 30 °C for 48 h for the enumeration of presumptive *B. cereus*, of which the presence was further confirmed using blood agar base (Oxoid) with addition of 7% of defibrinated sheep blood (E&O Labs, Bonnybridge, UK). Blood plates were incubated at 30 °C during 24 h. Streptomycin thallous acetate actidione agar (STAA; Oxoid) was incubated at 21 °C for 48 h for the enumeration of *B. thermosphacta*. The confirmation of *B. thermosphacta* was performed by both a catalase and oxidase test (Microbact Oxidase Strips; Oxoid). Oxytetracycline-glucose-yeast extract agar (OGYE; Oxoid) was incubated at 25 °C for 5 days for the enumeration of yeast and moulds. For PCA, OGYE, STAA, Slanetz and Bartley, MRS, M17, and VRBG agar, dilutions were used to prepare pour plates, which were incubated aerobically. For MRS, M17 and VRBG, an overlay of 10 mL was added. For RCA and TSC, dilutions were used to prepare pour plates, which were incubated anaerobically using AnaeroGen 3.5 L (Oxoid) in an airtight jar. For MYP agar, the spread-plating technique was used followed by aerobic incubation. The lower limit for enumeration for all media was 1 log colony forming unit (CFU)/g.

After making appropriate dilutions in MRD, the surface swab samples were plated on PCA for the enumeration of TAC, RCA for the enumeration of TANC and MRS agar for the enumeration of presumptive LAB, following the same incubation period and temperature as mentioned above.

Water activity (a_w_) was measured for each food sample using the AQUALAB 4TE water activity meter (Metergroup, München, Germany). The pH was measured using a pHenomal pH 2100 L in combination with a SF113 electrode (VWR, Leuven, Belgium).

### 2.3. Isolate Collection

For the collection of isolates, inoculated and incubated agar media used for the enumeration of TAC (PCA), total anaerobic count (RCA) and LAB (MRS agar) were used. On the agar media representing the highest decimal dilution showing bacterial growth, five to ten colonies were randomly picked from each of the three types of media, conforming to a median level of 31% of colonies available. Pure cultures were inoculated in brain heart infusion (BHI; Oxoid) for cultures originating from PCA and RCA or in MRS broth (Oxoid) for cultures originating from MRS agar, with 15% glycerol (Thermo Fisher Scientific, Geel, Belgium), incubated for 2 d at 21 °C and kept at −80 °C. From PCA, RCA and MRS agar, a total of 259, 227 and 197 isolates were collected, respectively.

### 2.4. Identification of Isolates

From each isolate, except for those that could not be cultivated after storage at −80 °C (128 out of 811 isolates), DNA was collected and stored at −20 °C. DNA was extracted according to Strandén et al. [21]. On these DNA extracts (GTG)_5_, PCR fingerprinting was carried out [22]. The obtained fingerprints were clustered using BioNumerics version 7.6 (Applied Maths, Sint-Martens-Latem, Belgium) based on their similarity using UPGMA (unweighted pair group method with arithmetic averages algorithm) with 1% curve smoothing. Out of 259, 227 and 197 isolates (originating from PCA, RCA and MRS agar, respectively), which were included in the (GTG)_5_ fingerprint clusters, respective totals of 147, 144 and 104 isolates were selected for identification. They were selected as representative isolates of visually defined clusters grouping isolates with the same band patterns. For clusters containing up to three isolates, one representative isolate was selected. A minimum of two isolates was selected for identification of clusters containing four or more isolates. A 1127 bp-region of the 16S rRNA gene was amplified for identification of the selected isolates, as previously described [21]. PCR products were Sanger-sequenced by a commercial service provider (Genewiz, Takeley, UK). The sequence reads were compared to the EZBioCloud database. To identify the isolates to their presumed species level, the species in the database were used with the highest identity (of at least 98.5%) and coverage. Biodiversity indexes were calculated by using Gleason–Margalef’s index [23].

From the slicers, 148 isolates were identified, including 58 isolates from PCA plates, 53 from RCA plates and 37 from MRS agar.

### 2.5. Metabarcoding

Ten grams of each sample were suspended into a 1:1 dilution using MRD. This suspension was gently kneaded to allow bacterial cells to detach from the matrix’s surface. Then, the suspension was centrifuged at 200× *g* for 3 min to remove potentially inhibiting food matrix material. The resulting supernatant was again centrifuged at 10,000 RCF for 10 min to pellet the bacterial cells. The resulting pellet was frozen using liquid N_2_ and stored at −80 °C prior to DNA extraction. DNA extraction was performed using the DNeasy mericon Food Kit (Qiagen, Antwerp, Belgium) following the manufacturer’s instructions. The DNA concentration was measured using the Quantus Fluorometer (Promega Benelux, Leiden, The Netherlands) in combination with the NanoPhotometer N60 (Implen, München, Germany). Amplicon sequencing of the V3–V4 region of the 16S rRNA gene was performed as described by the Illumina protocol and with primers of Klindworth et al. [24] on an Illumina MiSeq sequencer with 2 × 300 bp reads (Admera Health, South Plainfield, NJ, USA) The sequencing data have been deposited to the BioProject accession number PRJNA734172 in the NCBI SRA database.

The amplicon sequencing dataset was demultiplexed by the sequence provider, and barcodes were clipped off. The processing pipeline was entirely performed in RStudio 1.3.1093. Reads were imported into R and the ShortRead package was used to remove primer sequences [25]. Then, quality filtering and trimming of the reads were done using the “filterAndTrim” function from the DADA2 package [26]. Forward and reverse reads were trimmed to 280 bp and 210 bp, respectively, and quality filtering was performed with a maximum expected error of 2 for the forward and 4 for the reverse reads, after which the reads were merged and count tables from the amplicon sequence variants (ASVs) were calculated. Taxonomy was assigned using the RDP naive Bayesian classifier [27] with SILVA v138 as reference database. Rarefaction curves were made using the “rarecurve” function of the Vegan package. Shannon–Wiener diversity and Chao1 richness indices were calculated using the Phyloseq package. Seventeen cooked ham samples and 14 cooked chicken samples were not used in the downstream analyses because no plateau phase was reached in the rarefaction curves. The bacterial diversity was compared between conditions by calculating the Bray–Curtis dissimilarities between all samples and constructing non-metric multidimensional scaling plots (NMDS ordination plots) using phyloseq [28].

### 2.6. Statistical Analysis

Analysis of variance (ANOVA, confidence interval 95%) and Tukey’s test were performed using Version 1.3.1093 (R Core Team, R Foundation for Statistical Computing, Vienna, Austria) to verify whether enumerations were significantly different (*p*-value < 0.05).

## 3. Results

### 3.1. Microbiological Analysis

**Figure 2:**
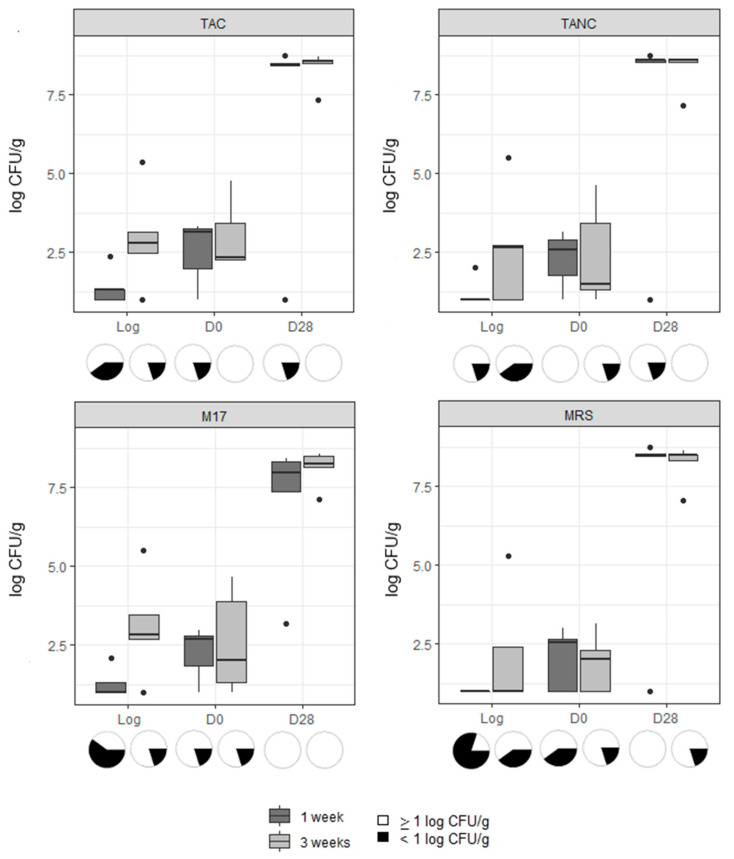
Log(CFU/g) values for total psychrotolerant and psychrophilic aerobic (TAC), and anaerobic counts (TANC) and lactic acid bacteria counts (M17 and MRS agar) found on cooked ham samples. The boxplots represent the 1st quartile, median, 3rd quartile and, if applicable, outliers of each dataset. Each boxplot contains five data points, except for when a sample was below the enumeration limit of 1 log CFU/g. In these cases, *n* will deviate. Deviation in data points is visualized by pie charts: white representing the countable samples and black representing the samples below the enumeration limit.

**Figure 3:**
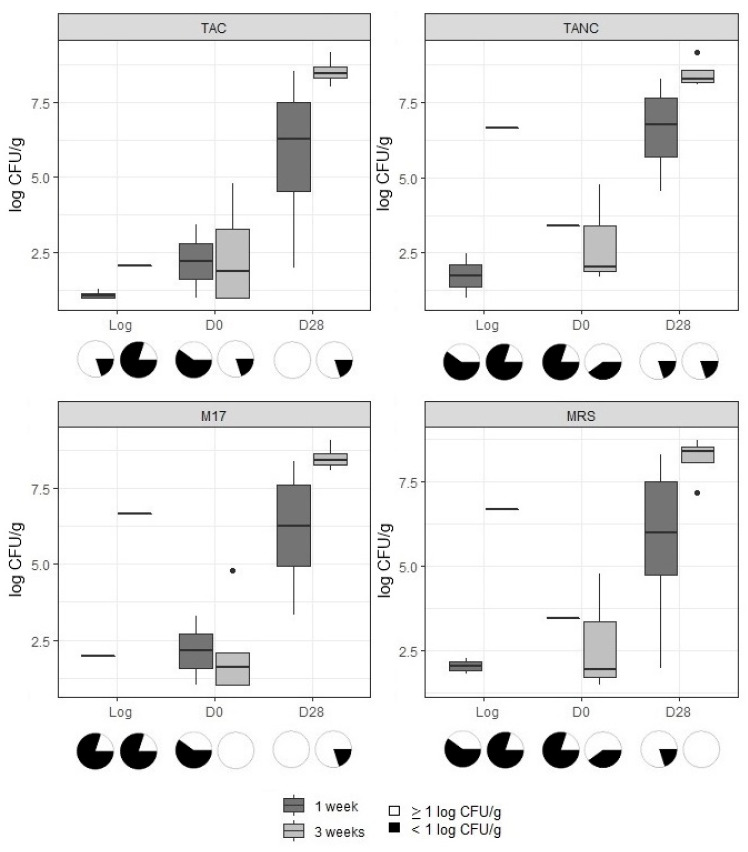
Log(CFU/g) values for total psychrotolerant and psychrophilic aerobic (TAC) and anaerobic counts (TANC) and lactic acid bacteria counts (M17 and MRS agar) found on cooked chicken samples. The boxplots represent the 1st quartile, median, 3rd quartile and, if applicable, outliers of each dataset. Each boxplot contains five data points, except for when a sample was below the enumeration limit of 1 log CFU/g. In these cases, *n* will deviate. Deviation in data points is visualized by pie charts: white representing the countable samples and black representing the samples below the enumeration limit.

The results for the microbiological enumerations (TAC, TANC and LAB) for cooked ham and cooked chicken were obtained (Figure 2 and Figure 3, respectively) and divided between samples taken of the 1-week old log, the 3-weeks old log and when these respective products were sliced and stored (see also Supplementary Table S1). Tukey’s test showed that, for each product type, there was no significant difference in bacterial counts between both storage times of the unsliced log and of the sliced products of logs sampled at day 0 and day 28, respectively. Additionally, for both product types, the counts for TAC, TANC and LAB (on both media; MRS and M17) were not significantly different at each analyzed production stage or sampling moment.

For cooked ham, the averages for TAC, TANC and LAB counted on MRS and M17 agar media varied between 7.1 and 8.6 log CFU/g at the end of the shelf-life period (Supplementary Table S1). Yeast and fungi were not often encountered; counts were below the enumeration limit in 70, 60 and 40% of the unsliced log, D0, and D28 samples, respectively. For cooked chicken samples, observations for TAC, TANC and LAB were largely similar, though average counts were slightly lower at the end of the shelf-life period than those of cooked ham, varying between 5.7 and 8.5 log CFU/g (Supplementary Table S1). Yeast and fungi were less often encountered in cooked chicken than in cooked ham samples. Despite the high levels of TAC, TANC and LAB at the end of the shelf-life period, only one sample (of cooked chicken) exhibited sensory changes at day 28, with visible slime formation and a sour smell. This was also the only sample of which counts for TAC, TANC and LAB on M17 agar exceeded 9 log CFU/g. Sulphite-reducing clostridia, Enterobacterales, *Bacillus cereus*, *Brochothrix thermosphacta* and *Enterococcus* spp. were rarely above the lower limit of enumeration (Table 1), but they occurred more often in the sliced cooked ham samples than in sliced cooked chicken.

Generally, slicing of the product had no immediate effect on pH and a_w_ (Table 1). In cooked ham, a reduction in pH at day 28 was found, dropping from approximately 6.2 to 5.7. Changes in pH at the end of the shelf-life period were less pronounced or absent in cooked chicken, and mainly occurred in the logs sliced at the end of their shelf-life period.

### 3.2. Identification

Isolates from cooked ham and cooked chicken samples were identified at species level and the results are shown in Table 2 and Table 3, respectively, with confidence intervals for the top-three (if three were present) most abundant identifications determined per type of sample.

For the cooked ham samples, a total of 425 colonies was identified. In the unsliced log samples, *B. thermosphacta* was the most abundant species representing 48, 68 and 50% of the isolates obtained from PCA, RCA and MRS agar, respectively. After slicing, there was more diversity noticeable, but *B. thermosphacta* still showed the highest abundance, especially among RCA isolates (17, 50 and 21% of the isolates from PCA, RCA and MRS agar, respectively). *Leuconostoc carnosum*, although not found in the unsliced log samples, was also relatively well-represented after slicing (7, 13 and 58% of the PCA, RCA and MRS agar isolates, respectively). At the end of the shelf-life period, species diversity was lower, and *L. carnosum* was clearly highly abundant (76, 71 and 93% of the PCA, RCA and MRS agar isolates, respectively). Also always present at the end of the shelf-life period, albeit to a lesser extent, was *Lactococcus piscium.* The latter species represented 12, 7 and 4% of the PCA, RCA and MRS agar isolates, respectively. In contrast, *B. thermosphacta* could no longer be recovered.

For the cooked chicken samples, a total of 258 isolates was identified at species level. *Latilactobacillus sakei* was highly abundant throughout the shelf-life period, although this was less clearly demonstrated on PCA (representing 13, 37 and 38% of the isolates obtained from the initial log and at day 0 and 28, respectively) than on RCA (100, 75 and 39%, respectively) and MRS agar (78, 78 and 48%, respectively). Carnobacteria also showed a clear presence throughout the shelf-life period, although a successive pattern of different species was found. *Carnobacterium inhibens* was only found on the unsliced logs (31 and 22% of the isolates from PCA and MRS isolates, respectively) and at day 0 (26 and 11% of the PCA and MRS isolates, respectively). *Carnobacterium maltaromaticum* was first identified at day 0, representing 25% of the RCA isolates, and was still present at the end of the shelf-life period (6, 15 and 5% of PCA, RCA and MRS isolates, respectively). *Carnobacterium divergens*, on the other hand, was mostly dominant at the end of the shelf-life period, representing 23, 12 and 24% of the PCA, RCA and MRS isolates, respectively. Prior to that, it was only found at day 0 on MRS agar (11% of the isolates).

**Figure 4:**
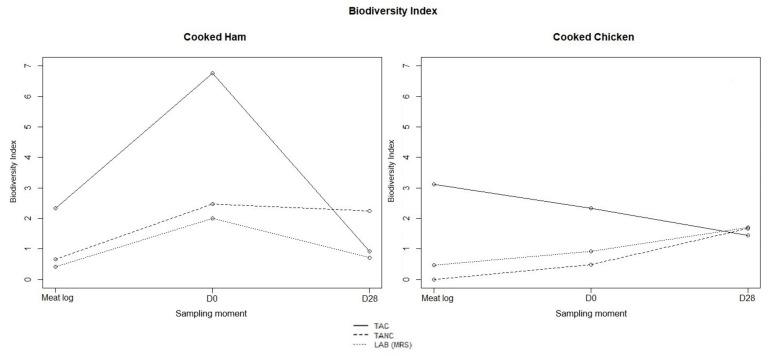
Biodiversity indexes for number of identified species found based on total psychrotolerant and psychrophilic aerobic (TAC) and anaerobic counts (TANC) and lactic acid bacteria counts (MRS agar) for cooked ham and cooked chicken at the different sampling moments.

The changes in biodiversity indexes between isolates obtained from the unsliced log as well as the samples at day 0 and 28 are summarized in Figure 4. For cooked ham, a biodiversity increase was seen when comparing the microbiota on the unsliced logs to the ones found on the samples at day 0. At the end of the shelf-life period, the acquired species diversity narrowed down, with certain species clearly dominating the microbiota (i.e., *L. carnosum* in cooked ham samples and *Lb. sakei* in cooked chicken samples).

Gram-negative bacteria were rarely present at the end of the shelf-life period. None of the isolates from cooked ham obtained for the samples at day 28 showed any presence of Gram-negative bacteria. For cooked chicken, *Serratia proteamaculans* was identified but represented only 3% of the isolates obtained from RCA.

Identities of surface sample isolates taken from the cooked ham slicer and the cooked chicken slicer were also determined (Supplemental Figures S1 and S2, respectively). As such, 74% of the identified isolates from the cooked ham slicer corresponded with species that were also found in the cooked ham product. They showed a predominant presence of *Enterococcus gilvus* and *Streptococcus parauberis*. Coincidentally, *E. gilvus* was largely found in the cooked ham log before slicing, while merely two colonies were identified as *S. parauberis* in the cooked ham samples, both right after slicing. *Brochothrix thermosphacta* was only identified once in the environmental samples, while this organism was highly present in the product log and right after slicing. Additionally, surface samplings indicated the presence of *C. divergens*, *C. maltaromaticum*, *L. carnosum*, *Psychrobacter maritimus*, *Psychrobacter faecalis* and *Vagococcus* spp., all of which were not or barely found in the unsliced log samples but could be identified during the later stage of the cooked ham storage.

For the cooked chicken slicer, 72% of the identified isolates were identified as species that were also found in the cooked chicken product. The surface samples indicated the presence of, primarily, *B. thermosphacta* and *Staphylococcus equorum*. In cooked chicken samples, *B. thermosphacta* only occurred at the end of the shelf-life period while *S. equorum* was found just once after slicing. *Carnobacterium divergens* and *C. maltaromaticum* were also found in both the environmental samples and the chicken samples. *Pseudomonas* spp., *Psychrobacter* spp. and *Enterococcus* spp., in contrast, were only identified in the environment and not in the cooked chicken samples.

### 3.3. Metabarcoding

**Figure 5:**
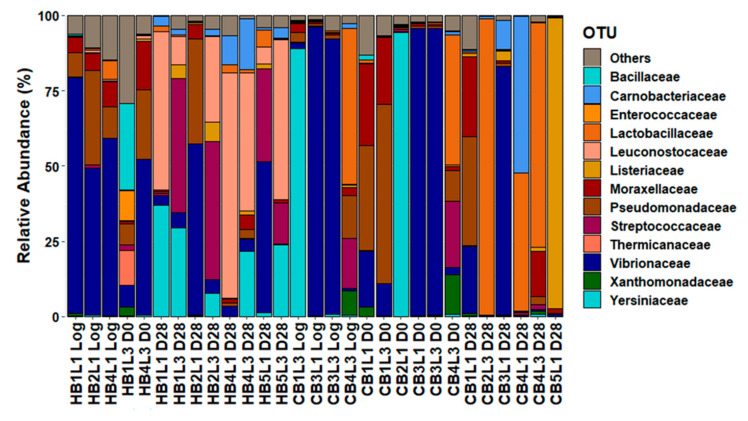
Cumulated histograms of the relative abundance of taxa identified by metabarcoding at family levels for cooked ham and cooked chicken samples. The taxa representing <5% in relative abundance were merged in the category “Others”. Sample codes: H, cooked ham; C, cooked chicken. B, batch number; L, time of slicing at either one (L1) or three weeks after log production (L3); and D, time of analysis at either the day of slicing (D0) or after 28 days of storage (D28).

**Figure 6:**
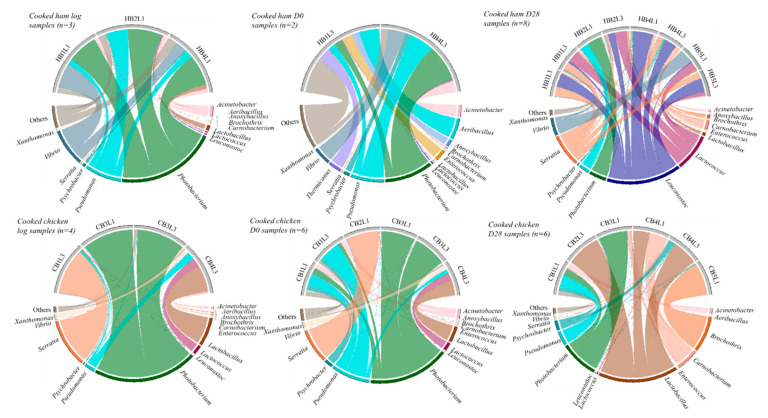
Chord diagrams of the relative abundance of taxa identified by metabarcoding at genus level for cooked ham and cooked chicken samples. The taxa representing <5% in relative abundance were merged in the category “Others”. Sample codes: H, cooked ham; C, cooked chicken; B, batch number; L, time of slicing at either one (L1) or three weeks after log production (L3); and D, time of analysis at either the day of slicing (D0) or after 28 days of storage (D28).

The metabarcoding analysis was performed on all samples of each product type (30 per type). Based on rarefaction curves, 13 cooked ham samples (3 of the unsliced log, 2 at day 0 and 8 at day 28) and 15 cooked chicken samples (3 of the unsliced log, 6 at day 0 and 6 at day 28) were retained for further analysis. The number of reads obtained per sample varied from 9051 to 341,236 reads, with an average of 81,489 reads per sample. Shannon–Wiener diversity and Chao1 richness indices are represented in Supplemental Figure S3. Figure 5 and Figure 6 show the relative population abundance at family and genus level, respectively. Early in the shelf-life period of both products, the genus *Photobacterium* was often present. In the cooked ham logs, its relative abundance varied between 43–49%; in cooked chicken logs, the relative abundance varied between 1–96%. The relative abundance of *Photobacterium* in samples from day 0 varied between 8–36% in the cooked ham samples and between 0–95% in the cooked chicken samples. At the end of the shelf-life period, the occurrence of *Photobacterium* was more rare, with a relative abundance below 5%, except in one of the eight analyzed cooked ham samples (57% relative abundance) and two of the six analyzed cooked chicken samples (22% and 83%).

In cooked ham, one genus other than *Photobacterium* was consistently found throughout the shelf-life period at a relative abundance of >5%, namely *Pseudomonas*. While the recovered taxa from the unsliced log samples showed clear similarities, the recovered taxa at day 0 of the two samples seemed more sample-specific with *Aeribacillus*, *Enterococcus* and *Thermicanus* being the most abundant taxa in one of the two analyzed samples and *Acinetobacter*, *Pseudomonas*, *Photobacterium* and *Vibrio* in the other. At the end of the shelf-life period, the genera *Leuconostoc*, *Serratia* and *Lactococcus* were most often recovered (i.e., in 7, 5 and 4 of the 8 analyzed samples and varying between 6–75%, 8–37% and 14–46%, respectively). Other more often recovered genera at D28 were *Carnobacterium* and *Vibrio*, each found in two samples.

The genera, besides *Photobacterium,* that were consistently recovered from the cooked chicken samples throughout the shelf-life period were reported as *Lactobacillus* (mainly found at day 28), and, similar to the cooked ham samples, *Pseudomonas* (mainly found at day 0). The taxa recovered and their abundance seemed sample-specific in cooked chicken, although similarities within batches could be found. Samples obtained from the unsliced logs in batch 3 and day-0 samples from batch 1 and 3 showed very similar recovered taxa and relative abundances within batches, while the other samples displayed different profiles. At the end of the shelf-life period, *Lactobacillus* was most often reported (in three of the six analyzed samples, at an abundance varying between 46–98%), followed by *Carnobacterium* (which was recovered in two samples at an abundance varying between 10–52%). *Brochothrix* was found in only one sample, but at the very high abundance of 96%.

Contrary to culture-dependent results, Gram-negative bacteria were more often recovered through metabarcoding analysis at the end of the shelf-life period. *Acinetobacter*, *Photobacterium*, *Pseudomonas*, *Serratia* and *Vibrio* were recovered in cooked ham samples at day 28, while cooked chicken samples from the same time point were characterized by the presence of *Acinetobacter*, *Photobacterium*, *Pseudomonas* and *Psychrobacter*.

## 4. Discussion

The microbiota of the two types of MAP cooked charcuterie products that were investigated in the present study were both dominated by LAB, whereas the counts of Gram-negative bacteria were relatively low. This is a common pattern for such products, when oxygen availability is low due to packaging [4,16,17,29]. In addition to this fairly trivial observation, more in-depth insights were obtained using a culture-dependent analysis, which were indicative of microbial interdependencies and related to the specificities of the products under study. *Latilactobacillus sakei*, for instance, is known to efficiently reduce the development of Enterobacterales in cooked ham [30], being a species that is cold-acclimatized and highly adapted to meat matrices [31,32]. In cooked chicken products, *Lb. sakei* was indeed typically dominant throughout the shelf-life period, in agreement with previous findings for MAP cooked poultry products [5,14], while Enterobacterales were not encountered. In contrast, *Lb. sakei* was only minimally present in cooked ham, whereas Enterobacterales were detectable in more than half of the investigated samples at the average level of 4 log CFU/g at the end of the shelf-life period. On the other hand, cooked ham samples were typified by the development of high levels of *L. carnosum* populations towards the end of the shelf-life period, in line with the fact that this is a commonly encountered species in such products [33,34]. Here, *Leuconostoc* spp. can be the cause of buttery aroma’s, slime formation, blowing of package and green discoloration [32,35]. Beyond their general acidifying effects, certain *L. carnosum* strains are known to display bioprotective potential, suppressing the growth of the background microbiota and limiting metabolite activity of *B. thermosphacta* in cooked ham environments [36]. Additionally, *Lc. piscium* was present, which has been shown to effectively reduce *B. thermosphacta* in cooked shrimp stored at 8 °C [37]. Be that as it may, presumable *B. thermosphacta* counts at the end of the shelf-life period of cooked ham were lower than for cooked chicken, where the presence of *L. carnosum* was minimal and *Lc. piscium* was not found.

*Carnobacterium* spp. were present in both products, usually throughout the shelf-life period. However, they were never able to manifest themselves to the same degree as was the case for *Lb. sakei* in cooked chicken or *L. carnosum* in cooked ham. Although carnobacteria, specifically *C. divergens* and *C. maltaromaticum*, are often described as meat spoilers when low-oxygen packaging is applied [32,38], they are less cold-adapted than *Leuconostoc* spp. and *Lactobacillus* spp. (or genera derived from *Lactobacillus* such as *Latilactobacillus*) and, therefore, less likely to prevail at temperatures below 12 °C [34]. An idiosyncratic finding for poultry was related to the high frequency in which *Carnobacterium inhibens* was identified in the unsliced log samples. This species is mostly recognized in relation to Atlantic salmon intestines, while carnobacteria in meat products usually belong to *C. divergens* and *C. maltaromaticum* [12,39,40]. The latter two species were nonetheless present at day 28.

Enterococci were only found in the samples derived from cooked ham, specifically at the end of the shelf-life period and in some product log samples. Enterococci can pose spoilage problems in cooked meats due to their ability to survive heating at 60 °C for 30 min [41,42]. Persistent presence of enterococci in cooked ham until the end of the shelf-life period has previously been reported, but only at elevated storage temperatures [43].

While the metabarcoding results were generally confirmative of the above-mentioned data obtained by the culture-dependent methods, for instance by identifying *Leuconostoc* and *Lactococcus* as the most abundant genera at the end of the shelf-life period, they also revealed some differences and added additional information. For instance, it was shown that most of the genera present, other than *Leuconostoc*, were more sample dependent than product dependent. In addition, the metabarcoding results only detected *Enterococcus* once with a significant relative abundance, while they yielded a high abundance of *Vibrio*, mainly found in the logs and at day 0, but also in some of the cooked ham samples from day 28. Raimondi et al. [13] also recovered *Vibrio*, which they further identified as *V. rumoiensis*, as a major component in cooked hams, mainly at the start of the shelf-life period. *Vibrio* spp. are normally considered marine bacteria, adapted to salty environments and commonly present on seafood products originating from warm water climates [44]. However, certain species still may show significant growth at temperatures below 7 °C [45].

The most revealing finding from the metabarcoding analysis, however, was that *Photobacterium* was often present in the unsliced logs and at day 0 for both the cooked ham and cooked chicken samples. Photobacteria have been found in meat products before when using culture-independent approaches [46,47,48,49,50], but usually they are more often associated with fish spoilage [51]. Numbers can be underestimated when general enumeration methods are used as a sufficiently high salt concentration in the growth medium is needed, and the bacteria can suffer heat injury when the pour plate technique is used [52]. Therefore, little is known at present about the spoilage-inducing abilities of photobacteria in meat products, although certain species are able to grow under anaerobic conditions at 0–20 °C, matching chilled MAP storage conditions [53]. Höll et al. [54] have predicted metabolic pathways in *Photobacterium* that could be relevant for spoilage and have found overlap with the metabolisms of the common meat spoilers *Brochothrix* and *Carnobacterium*. Even though *Photobacterium* was often recovered at the beginning of the shelf-life period, it nonetheless appeared to be outcompeted towards the end.

Both the culture-dependent and culture-independent techniques used in this study obviously come with their own set of advantages and disadvantages. One limit of the use of the former methodology is that certain viable organisms will not be cultivable under the conditions used, so that microorganisms such as *Photobacterium* risk being overlooked. Such microorganisms that are hard or impossible to cultivate with standard procedures can thus be recovered using the metabarcoding approach, resulting in a more complete overview of the product’s microbiota involved. A disadvantage, however, is that a reliable identification can only go down to genus level, and no distinction can be made between viable and non-viable bacteria. Another limit in this study, which affects both used methods, was the low abundance of microbiota present on the logs right before slicing. As enumerations in these types of samples are generally low, less information on the identity of the microbiota that are present can be amassed and certain bacteria can be overlooked when using the culture-dependent method. This is also an issue with the metabarcoding approach, as a high yield of pure DNA is desired after extraction to obtain reliable results, resulting in a large number of samples to be discarded after rarefaction curve analysis.

## Figures and Tables

**Table 1:** Values at three different production stages [unsliced log, after slicing (D0) and at end of the shelf-life period (D28)] for yeast and fungi (OGYE), sulphite-reducing clostridia (TSC), Enterobacterales (VRBG), presumptive *Bacillus cereus* (MYP), *Brochothrix thermosphacta* (STAA) and *Enterococcus* (Slanetz & Bartley), as well as the corresponding average (in bold) and standard deviation for pH and a_w_ values. For microbial counts, mean (in bold) and standard deviation log (CFU/g) values are given when there is more than one countable sample. In other instances, the log CFU/g value is given, in case of only one sample being countable, or the value is assigned as not detectable, when no samples were countable. The percentage of countable samples for each parameter was calculated based on the total number of samples taken at this specific production stage.

			OGYE		TSC		VRBG		MYP		STAA		Slanetz & Bartley	
	pH	a_w_	Countable (%)	Mean ± SD	Countable (%)	Mean ± SD	Countable (%)	Mean ± SD	Countable (%)	Mean ± SD	Countable (%)	Mean ± SD	Countable (%)	Mean ± SD
**Cooked ham**														
1 week														
Log	**6.18** ± 0.09	**0.9752** ± 0.0028	2 (40)	**1.0** ± 0.0	0 (0)	na	0 (0)	na	0 (0)	na	0 (0)	na	0 (0)	na
D0	**6.11** ± 0.16	**0.9759** ± 0.0037	2 (40)	**1.3** ± 0.5	0 (0)	na	0 (0)	na	0 (0)	na	1 (20)	1.9	0 (0)	na
D28	**5.73** ± 0.18	**0.9738** ± 0.0042	2 (40)	**2.0** ± 0.1	0 (0)	na	3 (60)	**4.4** ± 1.1	0 (0)	na	2 (40)	**4.2** ± 1.7	2 (40)	**5.5** ± 0.1
3 weeks														
Log	**6.17** ± 0.16	**0.9755** ± 0.0017	1 (20)	1.0	1 (20)	2.0	0 (0)	na	1 (20)	1.0	0 (0)	na	0 (0)	na
D0	**6.23** ± 0.15	**0.9756** ± 0.0033	2 (40)	**1.0** ± 0.0	0 (0)	na	0 (0)	na	0 (0)	na	0 (0)	na	1 (20)	1.3
D28	**5.74** ± 0.10	**0.9742** ± 0.0026	4 (80)	**2.4** ± 1.1	0 (0)	na	4 (80)	**3.9** ± 0.7	0 (0)	na	3 (60)	**4.1** ± 2.1	3 (60)	**5.7** ± 0.3
**Cooked chicken**														
1 week														
Log	**6.04** ± 0.25	**0.9727** ± 0.0019	0 (0)	Na ^a^	1 (20)	1.0	0 (0)	na	0 (0)	na	0 (0)	na	0 (0)	na
D0	**6.08** ± 0.08	**0.9735** ± 0.0021	0 (0)	na	0 (0)	na	0 (0)	na	0 (0)	na	0 (0)	na	0 (0)	na
D28	**6.04** ± 0.39	**0.9729** ± 0.0042	2 (40)	**1.6** ± 0.5	0 (0)	na	0 (0)	na	0 (0)	na	2 (40)	**6.2** ± 1.4	0 (0)	na
3 weeks														
Log	**6.21** ± 0.11	**0.9730** ± 0.0028	2 (40)	1.5 ± 0.7	0 (0)	na	0 (0)	na	0 (0)	na	0 (0)	na	0 (0)	na
D0	**6.18** ± 0.16	**0.9719** ± 0.0015	0 (0)	na	0 (0)	na	0 (0)	na	0 (0)	na	0 (0)	na	0 (0)	na
D28	**5.90** ± 0.40	**0.9711** ± 0.0043	2 (40)	**3.3** ± 2.3	0 (0)	na	0 (0)	na	1 (20)	1.0	1 (20)	8.1	0 (0)	na

^a^ Not applicable.

**Table 2:** Genus and presumptive species identity of isolates from PCA, RCA and MRS agar media for the cooked ham samples. Percentages of isolates were calculated on the total of identified isolates per medium. Confidence intervals for percentages of number of identified isolates are calculated for the three most abundant (if three were present) identifications per sampling moment from PCA, RCA and MRS agar media and are presented between square brackets.

Presumptive Species	Isolates from Cooked Ham (*n* = 425)								
	Log (*n* = 64)			D0 (*n* = 125)			D28 (*n* = 236)		
	PCA (%) *n* = 31	RCA (%) *n* = 21	MRS (%) *n* = 12	PCA (%) *n* = 54	RCA (%) *n* = 38	MRS (%) *n* = 33	PCA (%) *n* = 78	RCA (%) *n* = 86	MRS (%) *n* = 72
**Gram positive (*n* = 381)**									
*Arthrobacter glacialis*	0	0	0	1 (2)	0	0	0	0	0
*Arthrobacter psychrochitiniphilus*	1 (3)	0	0	0	0	0	0	0	0
*Brochothrix thermosphacta*	15 (48 [31–66])	15 (68 [49–88])	6 (50 [22–78])	9 (17 [9–27])	19 (50 [34–66])	7 (21 [7–35])	0	0	0
*Carnobacterium divergens*	0	0	0	0	2 (5)	1 (3)	1 (1)	3 (4)	0
*Carnobacterium maltaromaticum*	1 (3)	0	0	0	2 (5)	1 (3)	8 (10 [4–17])	5 (6 [1–11])	0
*Corynebacterium testudinoris*	0	0	0	2 (4)	0	0	0	0	0
*Enterococcus devriesei*	0	2 (11 [0–24])	0	0	1 (3)	0	0	0	0
*Enterococcus gilvus*	2 (7)	0	6 (50 [22–78])	0	0	0	0	2 (2)	0
*Enterococcus malodoratus*	0	0	0	0	0	1 (3)	0	0	1 (1)
*Latilactobacillus fuchuensis*	0	0	0	0	0	1 (3)	0	1 (1)	0
*Latilactobacillus sakei*	0	0	0	0	0	0	0	2 (2)	0
*Lactococcus piscium*	0	0	0	2 (4)	3 (8 [0–17])	2 (6 [0–14])	9 (12 [4–19])	6 (7 [2–12])	3 (4 [0–9])
*Leucobacter aridicollis*	1 (3)	0	0	0	0	0	0	0	0
*Leucobacter* spp.	2 (6)	0	0	0	0	0	0	0	0
*Leucobacter luti*	0	0	0	1 (2)	0	0	0	0	0
*Leuconostoc carnosum*	0	0	0	4 (7 [0–14])	5 (13 [2–24])	19 (58 [41–74])	59 (76 [66–85])	61 (71 [61–81])	67 (93 [87–99])
*Leuconostoc gelidum*	0	0	0	0	0	1 (3)	0	4 (5)	1 (1)
*Luteococcus japonicas*	0	0	0	0	2 (5)	0	0	0	0
*Microbacterium oxydans*	0	0	0	1 (2)	0	0	0	0	0
*Paeniglutamicibacter antarcticus*	0	0	0	1 (2)	0	0	0	0	0
*Staphylococcus saprophyticus*	0	0	0	2 (4)	0	0	0	0	0
*Streptococcus parauberis*	0	0	0	1 (2)	1 (3)	0	0	0	0
*Vagococcus fluvialis*	0	0	0	0	0	0	1 (1)	1 (1)	0
*Vagococcus salmoninarum*	0	0	0	0	1 (3)	0	0	0	0
*Vagococcus* spp.	0	0	0	0	0	0	0	1 (1)	0
**Gram negative (*n* = 44)**									
*Brevundimonas intermedia*	0	0	0	1 (2)	0	0	0	0	0
*Comamonas koreensis*	0	0	0	1 (2)	0	0	0	0	0
*Flavobacterium ardleyense*	0	0	0	3 (6)	0	0	0	0	0
*Limnohabitans planktonicus*	0	0	0	1 (2)	0	0	0	0	0
*Paracoccus aminophilus*	0	0	0	1 (2)	0	0	0	0	0
*Proteus terrae*	0	0	0	2 (4)	0	0	0	0	0
*Pseudochrobactrum spp.*	0	0	0	1 (2)	0	0	0	0	0
*Pseudomonas canadensis*	0	0	0	2 (4)	0	0	0	0	0
*Pseudomonas gessardii*	0	0	0	2 (2)	0	0	0	0	0
*Pseudomonas lactis*	0	0	0	1 (2)	0	0	0	0	0
*Pseudomonas psychrophila*	0	0	0	1 (2)	0	0	0	0	0
*Pseudomonas mandelii*	3 (10 [0–20])	0	0	0	0	0	0	0	0
*Pseudomonas thivervalensis*	1 (3)	0	0	0	0	0	0	0	0
*Psychrobacter faecalis*	0	0	0	1 (2)	0	0	0	0	0
*Psychrobacter maritimus*	0	0	0	8 (15 [5–24])	0	0	0	0	0
*Psychrobacter pulmonis*	0	0	0	1 (2)	0	0	0	0	0
*Rhizobium radiobacter*	0	0	0	1 (12)	0	0	0	0	0
*Serratia myotis*	5 (16 [3–29])	4 (21 [4–39])	0	0	2 (5)	0	0	0	0
*Shewanella putrefaciens*	0	0	0	1 (2)	0	0	0	0	0
*Sphingobacterium anhuiense*	0	0	0	1 (2)	0	0	0	0	0
*Sphingobacterium faecium*	0	0	0	1 (2)	0	0	0	0	0

**Table 3:** Genus and presumptive species identity of isolates from PCA, RCA and MRS agar media for the cooked chicken samples. Percentages of isolates were calculated on the total of identified isolates per medium. Confidence intervals for percentages of number of identified isolates are calculated for the three most abundant (if three were present) identifications per sampling moment from PCA, RCA and MRS agar media and are presented between square brackets.

Presumptive Species	Isolates from Cooked Chicken (*n* = 258)								
	Log (*n* = 30)			D0 (*n* = 36)			D28 (*n* = 192)		
	PCA (%) *n* = 13	RCA (%) *n* = 8	MRS (%) *n* = 9	PCA (%) *n* = 19	RCA (%) *n* = 8	MRS (%) *n* = 9	PCA (%) *n* = 64	RCA (%) *n* = 66	MRS (%) *n* = 62
**Gram positive (*n* = 247**)									
*Bacillus licheniformis*	1 (8)	0	0	0	0	0	0	0	0
*Brochothrix thermosphacta*	0	0	0	0	0	0	14 (22 [12–32])	16 (24 [14–35])	6 (10 [2–17])
*Carnobacterium divergens*	0	0	0	0	0	1 (11)	15 (23 [13–34])	8 (12 [7–24])	15 (24 [14–35])
*Carnobacterium inhibens*	4 (31 [6–56])	0	2 (22 [0–49])	5 (26 [7–46])	0	1 (11)	0	0	0
*Carnobacterium maltaromaticum*	0	0	0	0	2 (25 [0–55])	0	4 (6)	10 (15)	3 (5)
*Enterococcus malodoratus*	0	0	0	0	0	0	0	0	1 (2)
*Enterococcus viikkiensis*	0	0	0	0	0	0	1 (2)	0	0
*Kocuria rhizophila*	0	0	0	1 (5)	0	0	0	0	0
*Latilactobacillus sakei*	2 (13 [0–35])	8 (100 [100])	7 (78 [51–100])	7 (37 [15–59])	6 (75 [45–100])	7 (78 [51–100])	24 (38 [26–49])	27 (41 [28–51])	30 (48 [36–61])
*Leuconostoc carnosum*	0	0	0	0	0	0	1 (2)	1 (2)	1 (2)
*Micrococcus caseolyticus*	1 (8)	0	0	0	0	0	0	0	0
*Staphylococcus spp.*	0	0	0	1 (5)	0	0	0	0	0
*Staphylococcus sciuri*	0	0	0	1 (5)	0	0	0	0	0
*Vagococcus* spp.	0	0	0	0	0	0	0	0	1 (2)
*Vagococcus silagei*	0	0	0	0	0	0	5 (8)	2 (3)	5 (8)
**Gram negative (*n* = 11)**									
*Acinetobacter albensis*	0	0	0	2 (11 [0–24])	0	0	0	0	0
*Comamonas* spp.	0	0	0	1 (5)	0	0	0	0	0
*Pseudomonas migulae*	1 (8)	0	0	0	0	0	0	0	0
*Pseudomonas weihenstephanensis*	1 (8)	0	0	0	0	0	0	0	0
*Psychrobacter maritimus*	0	0	0	1 (5)	0	0	0	0	0
*Ralstonia mannitolilytica*	1 (8)	0	0	0	0	0	0	0	0
*Serratia proteamaculans*	0	0	0	0	0	0	0	2 (3)	0
*Sphingobacterium cladoniae*	1 (8)	0	0	0	0	0	0	0	0
*Sphingobacterium pakistanense*	1 (8)	0	0	0	0	0	0	0	0

## Data Availability

Raw 16S sequence reads are available at the Sequence Read Archive (SRA: https://www.ncbi.nlm.nih.gov/sra (accessed on 14 May 2021)) under the BioProject PRJNA734172.

## Supplementary Materials

The following are available online at https://www.mdpi.com/article/10.3390/microorganisms9061223/s1, Figure S1: Genus and species identity of isolates from the cooked ham slicer, Figure S2: Genus and species identity of isolates from the cooked chicken slicer, Figure S3: Chao1 and Shannon-Wiener diversity richness indices, Table S1: TAC, TANC and LAB counts at different production and sampling stages.
