# Supplementary material for: The Microbiota of Modified-Atmosphere-Packaged Cooked Charcuterie Products throughout Their Shelf-Life Period, as Revealed by a Complementary Combination of Culture-Dependent and Culture-Independent Analysis
Source: Microorganisms. 2021 Jun 4;9(6):1223. doi: 10.3390/microorganisms9061223 (PMC8229102; doi:10.3390/microorganisms9061223)
Supplement: Supplementary file 1 [file microorganisms-09-01223-s001.zip › microorganisms-1241226-supplementary/Supplemental material/Chao1 and Shannon-Wiener diversity richness indices.pdf]

Supplemental material.

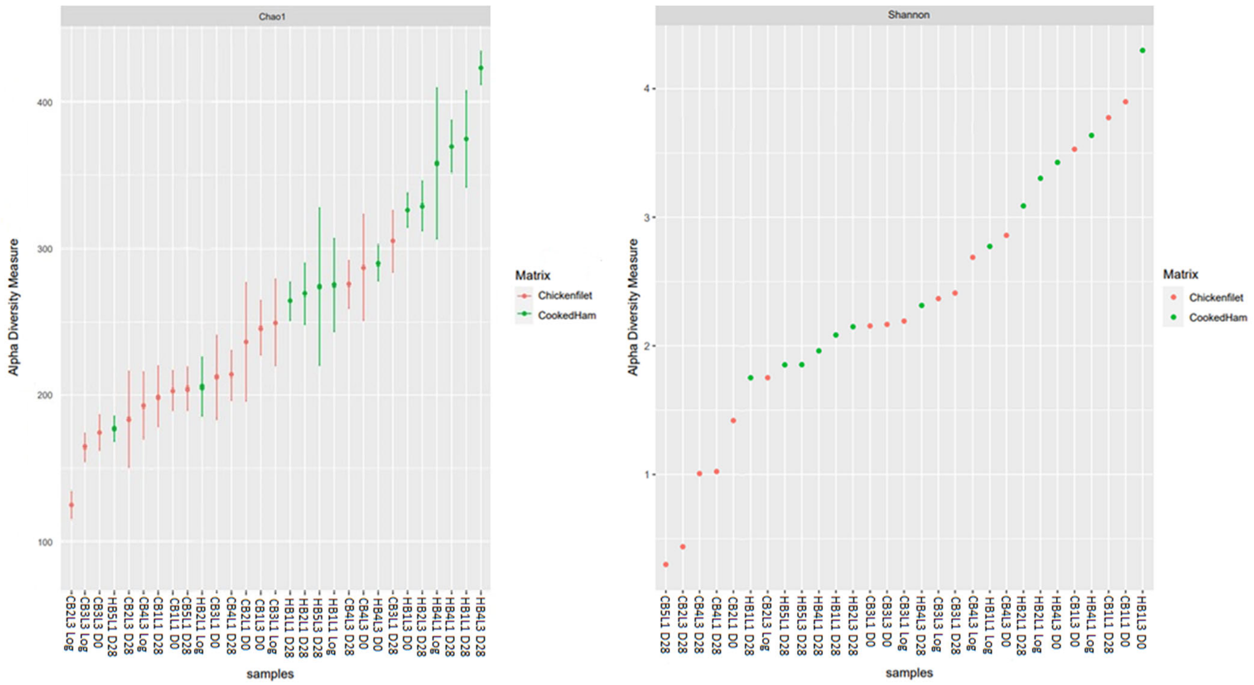

Figure S3: Chao1 and Shannon-Wiener diversity richness indices. These indices represent the alpha diversity of taxa within the samples. When a sample has a high alpha diversity, it means many different organisms are present in the sample.
