# Supplementary material for: The Microbiota of Modified-Atmosphere-Packaged Cooked Charcuterie Products throughout Their Shelf-Life Period, as Revealed by a Complementary Combination of Culture-Dependent and Culture-Independent Analysis
Source: Microorganisms. 2021 Jun 4;9(6):1223. doi: 10.3390/microorganisms9061223 (PMC8229102; doi:10.3390/microorganisms9061223)
Supplement: Supplementary file 1 [file microorganisms-09-01223-s001.zip › microorganisms-1241226-supplementary/Supplemental material/Genus and species identity of isolates of the food contact surface swab samples obtained from the cooked ham slicer.pdf]

Supplemental material.

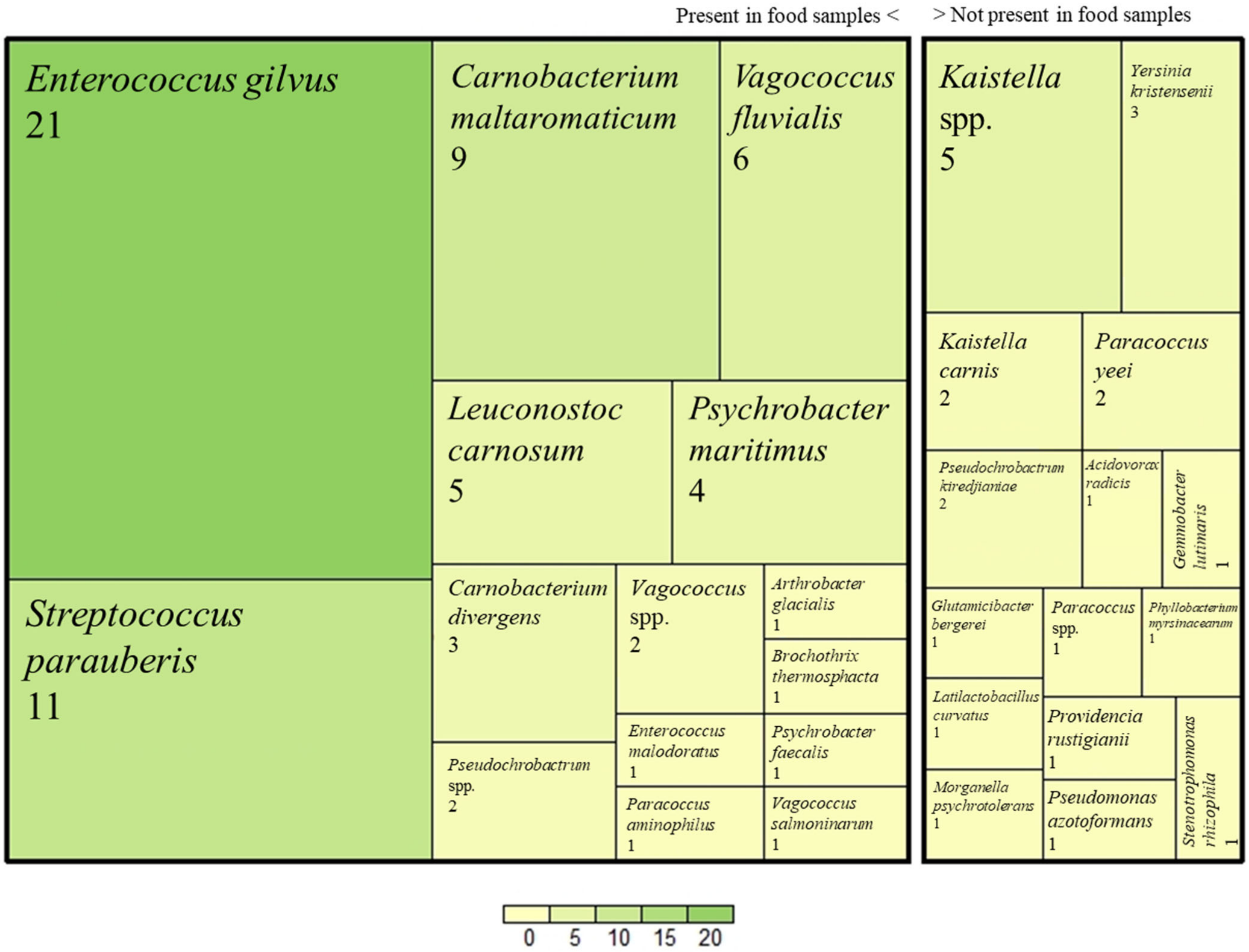

Figure S1: Genus and species identity of isolates (n=95) from PCA, RCA and MRS agar media of the food contact surface swab samples obtained from the cooked ham slicer.
