# Supplementary material for: The Microbiota of Modified-Atmosphere-Packaged Cooked Charcuterie Products throughout Their Shelf-Life Period, as Revealed by a Complementary Combination of Culture-Dependent and Culture-Independent Analysis
Source: Microorganisms. 2021 Jun 4;9(6):1223. doi: 10.3390/microorganisms9061223 (PMC8229102; doi:10.3390/microorganisms9061223)
Supplement: Supplementary file 1 [file microorganisms-09-01223-s001.zip › microorganisms-1241226-supplementary/Supplemental material/VALUES~1.pdf]

Supplemental material.

Table S1: Values at different production and sampling stages [unsliced log, after slicing (D0) and at end of shelf life (D28)] for values of total psychrotolerant and psychrophilic aerobic (TAC) and anaerobic counts (TANC) and for lactic acid bacteria counts (LAB). For microbial counts, means (in bold) and standard deviations are given as log(CFU/g) values when there was more than one countable sample. In other instances, the log(CFU/g) value is given of the only countable sample. The percentage of countable samples for each parameter was calculated based on the total number of samples taken at this specific sampling stage.

	**TAC**		**TANC**		**LAB (MRS)**		**LAB (M17)**	
Sample type	Countable (%)	Mean ± SD	Countable (%)	Mean ± SD	Countable (%)	Mean ± SD	Countable (%)	Mean ±SD
Cooked ham								
1 week								
Log	3 (60)	**1.7** ± 0.6	1 (20)	2.0	1 (20)	1.0	2 (40)	**1.7** ± 0.6
D0	4 (80)	**2.9** ± 0.6	5 (100)	**2.3** ± 0.9	3 (60)	**2.7** ± 0.2	4 (80)	**2.6** ± 0.5
D28	4 (80)	**8.5** ± 0.2	4 (80)	**8.6** ± 0.1	4 (80)	**8.6** ± 0.1	5 (100)	**7.1** ± 2.2
3 weeks								
Log	4 (80)	**3.4** ± 1.3	3 (60)	**3.6** ± 1.6	3 (60)	**2.9** ± 2.2	4 (80)	**3.6** ± 1.3
D0	5 (100)	**3.0** ± 1.1	4 (80)	**2.7** ± 1.6	4 (80)	**2.1** ± 0.9	4 (80)	**3.0** ± 1.6
D28	5 (100)	**8.3** ± 0.6	5 (100)	**8.3** ± 0.7	5 (100)	**8.2** ± 0.7	5 (100)	**8.1** ± 0.6
Cooked chicken								
1 week								
Log	4 (80)	**1.4** ± 0.5	2 (40)	**1.7** ± 1.0	2 (40)	**2.1** ± 0.3	1 (20)	2.0
D0	2 (40)	**2.2** ± 1.7	1 (20)	3.4	1 (20)	3.5	2 (40)	**2.1** ± 1.6
D28	5 (100)	**5.8** ± 2.6	4 (80)	**6.6** ± 1.6	4 (80)	**5.7** ± 2.5	5 (100)	**6.1** ± 2.0
3 weeks								
Log	1 (20)	2.1	1 (20)	6.6	1 (20)	6.7	1 (20)	6.7
D0	4 (80)	**2.4** ± 1.8	3 (60)	**2.8** ± 1.7	3 (60)	**2.7** ± 1.8	5 (100)	**2.1** ± 1.6
D28	4 (80)	**8.5** ± 0.5	4 (80)	**8.5** ± 0.5	4 (80)	**8.2** ± 0.7	4 (80)	**8.5** ± 0.4
